# Biostimulants for Plant Growth and Mitigation of Abiotic Stresses: A Metabolomics Perspective

**DOI:** 10.3390/metabo10120505

**Published:** 2020-12-10

**Authors:** Lerato Nephali, Lizelle A. Piater, Ian A. Dubery, Veronica Patterson, Johan Huyser, Karl Burgess, Fidele Tugizimana

**Affiliations:** 1 Research Centre for Plant Metabolomics, Department of Biochemistry, University of Johannesburg, Auckland Park, Johannesburg 2006, South Africa; leratopertu@yahoo.com (L.N.); lpiater@uj.ac.za (L.A.P.); idubery@uj.ac.za (I.A.D.); 2 International Research and Development, Omnia Group, Johannesburg 2191, South Africa; Veronica.Patterson@omnia.co.za (V.P.); Johan.Huyser@omnia.co.za (J.H.); 3 Institute of Quantitative Biology, Biochemistry and Biotechnology, School of Biological Sciences, University of Edinburgh, Edinburgh EH8 9AB, UK; Karl.Burgess@ed.ac.uk

**Keywords:** abiotic stresses, biostimulants, food security, metabolomics, growth-promoting rhizobacteria (PGPR), plant defenses

## Abstract

Adverse environmental conditions due to climate change, combined with declining soil fertility, threaten food security. Modern agriculture is facing a pressing situation where novel strategies must be developed for sustainable food production and security. Biostimulants, conceptually defined as non-nutrient substances or microorganisms with the ability to promote plant growth and health, represent the potential to provide sustainable and economically favorable solutions that could introduce novel approaches to improve agricultural practices and crop productivity. Current knowledge and phenotypic observations suggest that biostimulants potentially function in regulating and modifying physiological processes in plants to promote growth, alleviate stresses, and improve quality and yield. However, to successfully develop novel biostimulant-based formulations and programs, understanding biostimulant-plant interactions, at molecular, cellular and physiological levels, is a prerequisite. Metabolomics, a multidisciplinary omics science, offers unique opportunities to predictively decode the mode of action of biostimulants on crop plants, and identify signatory markers of biostimulant action. Thus, this review intends to highlight the current scientific efforts and knowledge gaps in biostimulant research and industry, in context of plant growth promotion and stress responses. The review firstly revisits models that have been elucidated to describe the molecular machinery employed by plants in coping with environmental stresses. Furthermore, current definitions, claims and applications of plant biostimulants are pointed out, also indicating the lack of biological basis to accurately postulate the mechanisms of action of plant biostimulants. The review articulates briefly key aspects in the metabolomics workflow and the (potential) applications of this multidisciplinary omics science in the biostimulant industry.

## 1. Introduction

Plants are continuously exposed to biotic (e.g., pest and pathogen attacks) and abiotic stresses (e.g., drought, extreme temperatures and salinity), which lead to frequent adjustment and remodeling of the plant defense machinery, as well as involving reconfiguration of the plant metabolism [1,2]. Evolutionarily, plants have developed a multilayered and dynamic defense system that renders them as adept as animals in responding effectively to ever-changing environments [3]. Furthermore, plant defense strategies can be enhanced and sensitized using various approaches such as biological and chemical priming. Defense priming is a phenomenon whereby the plant immune system and abiotic defenses are preconditioned, resulting in faster, stronger and effective defense and resistance mechanisms against subsequent biotic and abiotic stresses [4,5,6]. This immune-stimulation of plants, which is postulated to be an adaptive and low-cost defensive measure, is a result of interactions of plants with beneficial microbes, chemical compounds, insect herbivores or environmental cues [7,8]. 

There is emerging evidence of biostimulants as plant priming agents, as demonstrated by the observed effectiveness of these formulations in promoting and sensitizing plant defenses and resistance against different environmental stresses [9]. In the last decade, the field of plant biostimulants has been steadily growing in the agricultural industry and has positioned itself as one of the key emerging strategies for enhancing crop production and resilience to the changing climate. Plant biostimulants have received considerable attention lately, and are increasingly being integrated into agriculture and production systems as plant growth and yield regulators/promoters as well as pre-stress conditioners [10]. However, the limited fundamental research into the modes of action of many biostimulant products is among the knowledge gaps that require scientific attention. Elucidation of the biological basis of biostimulant function, and a broad mechanism of action at a cellular and molecular levels, is a prerequisite for the development of a scientifically-based biostimulant industry, leading to an effective exploration and application of formulations in agriculture [9,10]. Few emerging studies have shown the impeccable capabilities and potential of metabolomics approaches to reveal the mechanistic insights describing biostimulant-plant interactions [11,12]. Furthermore, these studies have proven that metabolomics studies can provide key fundamental knowledge and understanding required to explore novel biostimulant-based strategies for improved crop health and resilience in a changing climate. 

Metabolomics is classically defined as a comprehensive and holistic measurement of the entire complement of small molecular weight molecules, namely metabolites (≤1500 Da in size), within a biological system [13]. The metabolome, being the chemical space and language of metabolism, carries imprints of genetic and environmental factors, and is expectedly more sensitive to perturbations in both metabolic fluxes and enzyme activity than either the transcriptome or proteome [14,15]. Hence, the global quantitative measurements of the metabolome provide an exploration of small cellular worlds, revealing hidden patterns and (predictively) reflecting functional signatures of the biochemical landscape and cellular physiology of the system under consideration [13,16,17]. Applying this multidisciplinary omics science in the plant biostimulant field would thus generate integrated knowledge that sheds light on modes and mechanisms of action of biostimulants at cellular and molecular levels. Thus, this review provides an overview of plant defence strategies against biotic and abiotic stresses, and reports on current definitions, claims and applications of plant biostimulants, also pointing out the lack of biological basis to accurately postulate or articulate the mechanisms of action of plant biostimulants. Furthermore, this review highlights some key aspects in the metabolomics workflow and some recent advancements in the field; and lastly, we discuss the applications of metabolomics in the investigation of biostimulant–plant interactions under adverse environmental conditions.

## 2. Basic and Overlapping Framework of Plant Immunity and Defense against Biotic and Abiotic Stresses

In their natural habitats, plants coexist with highly dynamic microbial communities, some of which are harmful to plant health. Furthermore, the environmental constraints such as drought, salt and extreme temperatures negatively affect plant growth and development, both at the cellular and organismal levels [18,19]. Thus, for optimal growth and development, plants must have a protective immune and defense system that properly integrates both microbial signals and abiotic factors at both local and systemic levels. Studies have revealed that, evolutionarily, plants have developed constitutive, active, inducible and tightly regulated immune and defense systems that mediate interactions with heterogeneous environments comprising both biotic and abiotic stresses. The result of these dynamic and complex interactions is a determining factor for plant survival and fitness [20,21]. Several models have been proposed for describing plant immune responses to biotic stresses, with a common denominator that the innate immune system is based on the surveillance and perception of non-self, damaged-self and altered-self, broadly termed “danger” signals [22,23]. Generally, the first line of defense in plant response to biotic stresses involves preformed physical and chemical barriers such as cutin and waxes (cuticles), cell walls, antimicrobial enzymes and secondary metabolites to prevent or attenuate invasion of various pathogens [23,24]. In a case of a successful pathogen entry or plant alteration and/or damage, the second line of defense is launched, triggered by the recognition of danger signals through structurally diverse transmembrane or intracellular pattern recognition receptors (PRRs). This recognition of danger signals activates several levels of induced defense responses through a complex network of signal transduction and amplification, both locally at the site of infection and systemically in distant tissues [22,25]. 

The first layer of these inducible defenses is put in motion through the perception of microbe/pathogen-associated molecular patterns (M/PAMPs) or damage-associated molecular patterns (DAMPs) and termed M/PAMP-triggered immunity (M/PTI). The latter involves a series of complex cellular and molecular reprogramming events that are translated into distinct biochemical and physiological phenomena, such as activation of a signaling network involving phytohormones and other signaling molecules, stomatal closure to stop pathogen penetration, production of reactive oxygen species (ROS) and nitric oxide (NO), callose deposition and the de novo biosynthesis of antimicrobial metabolites due to reconfiguring of cellular metabolism [21,26]. However, due to the coevolution of plant-pathogen interactions, specialized pathogens have developed means to suppress M/PTI, through a repertoire of effector molecules that are translocated into the plant cell where they can alter cellular metabolism and homeostasis to promote disease. This state is known as effector-triggered susceptibility (ETS). On the other hand, plants have evolved mechanisms to perceive these effectors through intracellular receptors known as resistance (R) proteins. The recognition of these effectors activates the second layer of defense known as effector-triggered immunity (ETI). One of the descriptive markers of ETI events is the hypersensitive response (HR), which is an induced localized programmed cell death, to limit the spread of pathogen infection [21,23,26].

Studies have shown that there is an interplay (or overlap) between M/PTI and ETI, which is explained by the convergence between signaling and downstream biochemical events induced by both layers of defenses. This highly regulated coordination of M/PTI- and ETI-related defenses defines the basal defensive metabolism and subsequent physiological state of the plant against pathogen attack [22,27]. Furthermore, locally induced defense responses can trigger a defensive state in distant parts of the plant, a phenomenon known as systemic acquired resistance (SAR). The latter confers long-lasting protection against a range of pathogens in the systemic healthy tissue of plants undergoing a localized pathogen infection. Another form of an induced resistance mechanism is induced systemic resistance (ISR). The latter results from the interactions of plants and beneficial microbes, such as plant growth-promoting rhizobacteria and fungi [28,29]. Elaborated details of cellular and molecular events underlying these layers and models of plant immune responses to biotic stresses are beyond the scope of this review but can be found in the literature cited herein. 

**Figure 1:**
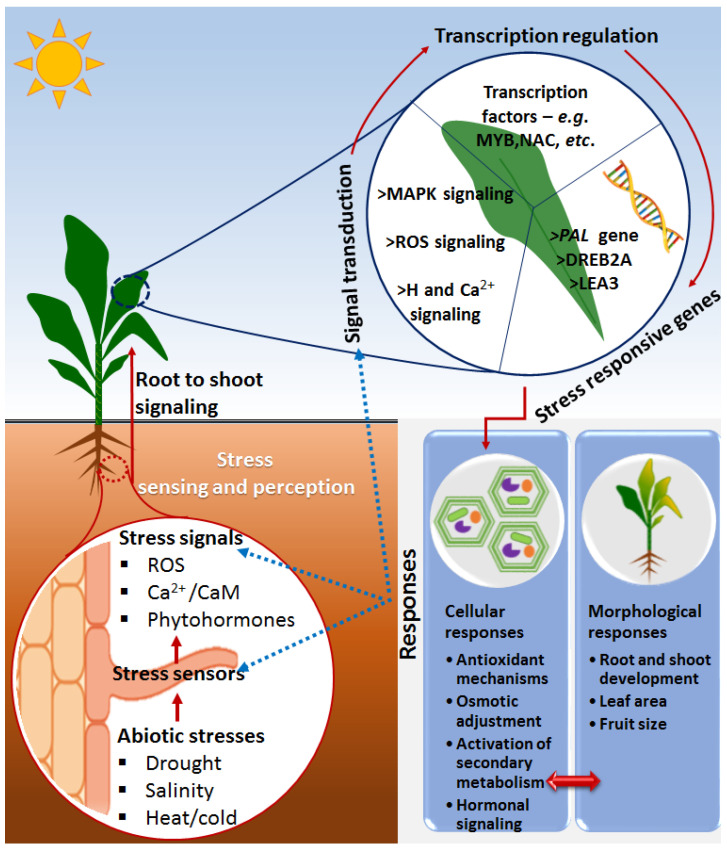
**A descriptive model showing some general molecular events in a plant responding to abiotic stresses**. Upon the perception of stress signals via evolutionarily developed rapid-sensing mechanisms, the systematic signal transduction/pathways such as ROS, Ca^2+^ and phytohormone signals are activated, leading to regulation of stress-responsive genes as facilitated by transcription factors. The stress-related genes are (de)activated to orchestrate the induction of stress adaptation/resistance cellular mechanisms such as antioxidant mechanisms, osmotic adjustment and secondary metabolism adjustment, as well as morphological responses such as root and shoot development, leaf area and fruit size. The blue dotted lines indicate the influence of the stress responses to sensors, signals and signal transductions. Abbreviations: Ca^2+^ = calcium ion, Ca^2+^/CaM = calcium calmodulin, ROS = reactive oxygen species, MAP kinases = mitogen-activated protein kinase, H = hormone, MYB = myeloblastosis, NAC = non-amyloid-β component, PAL = phenylalanine ammonia-lyase, DREB2A = dehydration-responsive element-binding protein 2A, LEA3 = late embryogenesis abundant 3.

Plants are not only exposed to harmful microorganisms but also to harsh environmental conditions such as drought, salinity, extreme temperatures and nutrient deficiency, which adversely affect plant growth and productivity [30,31]. Some of the general defense responses include a highly-regulated cascade of signal transduction events, activation of defense-related genes, the accumulation of ROS and antioxidant mechanisms, production of defense-related metabolites and subsequent physiological and morphological changes [32]. Furthermore, one of the primary events in the plant responses to abiotic stresses involves a highly regulated and coordinated web of plant hormones such as abscisic acid (ABA), auxins (indole acetic acid, IAA), ethylene (ET), cytokinins (CK), gibberellins (GA), salicylic acid (SA), jasmonic acid (JA) and brassinosteroids (BRs). These phytohormones often act as primary signaling molecules that trigger complex signaling cascades, subsequently leading to stimulation of the expression of stress-related genes and induction of physiological and morphological changes, which eventually lead to abiotic stress tolerance or resistance (Figure 1) [33,34,35]. 

Some of the common physiological responses to major abiotic stresses include a reduction in both transpiration and photosynthesis rates, a decrease in stomatal conductance and in leaf water content and a reduced relative growth rate [36,37]. For instance, responding to drought stress, molecular and cellular events deployed by the plant are translated into a reconfiguration of plant cellular metabolism, similar to defense responses to biotic stress. The latter leads to physiological modulation for plant survival. Some of the reported defense-related morpho-physiological changes due to drought stress are root swelling to promote nutrient uptake, leaf stomatal closure and leaf rolling to prevent water loss via transpiration, decreased chlorophyll content, leaf abscission and decrease in leaf area contributing to the reallocation of nutrients stored in older and diseased leaves to new leaves or the shoot [32,38]. 

Growth arrest is also one of the primary effects of drought stress. The inhibition of shoot growth is known to minimize the metabolic demands under water stress, thus increasing the biosynthesis and assimilation of metabolites such as osmolytes. The latter, also known as osmoprotectants, are low molecular weight, soluble compounds that play fundamental roles in osmotic adjustment, consequently conferring protection against cell drying-out. Osmoprotectants efficiently maintain osmotic balance, and stabilize membranes and macromolecules under water stress conditions. These include betaines, amino acids, polyols and non-reducing sugars (e.g., glycine betaine, proline and inositol) [39]. Root growth arrest maintains the function of root meristem and promotes rapid growth once the stress has been alleviated [40]. The inhibition of lateral root growth was also observed as an adaptive mechanism that promotes primary root elongation to reach the water from lower layers in the soil [41]. The other key cellular event that is observed under both biotic and abiotic stresses is the perturbations in the highly-regulated ROS homeostasis, causing an over-accumulation of ROS such as singlet oxygen (^1^O_2_), superoxide anion radical (O_2_^•−^), hydroxyl radical (^•^OH) and hydrogen peroxide (H_2_O_2_). The accumulation of these species results from the imbalance between the ROS production and the effectiveness of ROS scavenging systems, subsequently causing an oxidative burst and adversely affecting the plant metabolism by damaging cellular components such as DNA, proteins and lipids [42,43,44]. ROS generation can be detrimental as well as advantageous; it can also serve as a biological marker during abiotic stress conditions and can induce stress-signaling pathways to inhibit further damages [43,45]. To effectively manage and alleviate the oxidative stress, plants utilize ROS scavenging systems comprising (i) the enzymatic antioxidant system and (ii) a non-enzymatic antioxidant mechanism also known as the “low molecular weight” antioxidant system [45,46].

**Figure 2:**
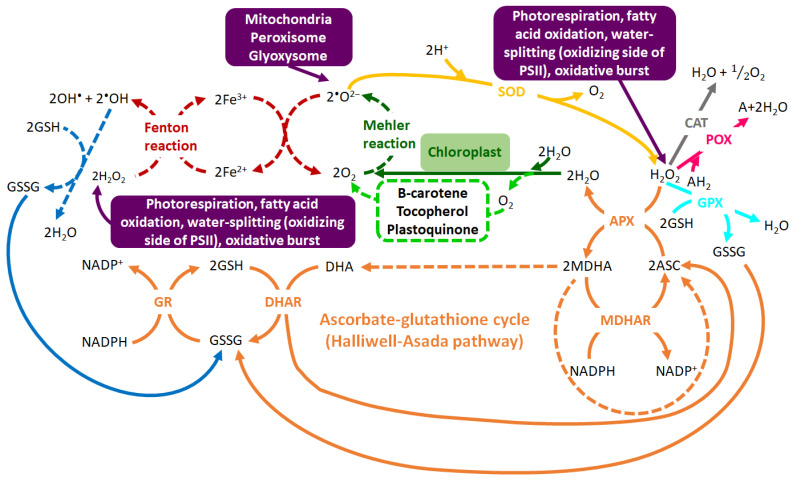
**The intricate web of the highly-regulated oxidant-antioxidant system**. Hydroxyl radical (^•^OH) and superoxide anion radical (^•^O_2_^−^) are generated via the Fenton reaction (in red). ^•^O_2_^−^ is produced by Mehler reaction (in dark green) and singlet oxygen (^1^O_2_) from water molecules (in neon green). The initial step in the ROS scavenging is the conversion of ^•^O_2_^−^ into hydrogen peroxide (H_2_O_2_) by superoxide dismutase SOD (in yellow), followed by the detoxification of H_2_O_2_ by GPX (in turquoise), CAT (in grey), APX (in orange) and POX (in pink). H_2_O_2_ is also scavenged through the ascorbate-glutathione cycle (in orange) which uses ASC and GSH as the cyclic transfer of reducing equivalents and NADPH as reducing power. The removal of ^•^OH by GSH (in blue) forms GSSG which is regenerated to GSH via the GR-mediated reaction. The enzymatic pathways are depicted by bold lines and non-enzymatic pathways are indicated by dotted lines. Abbreviations: GPX = glutathione peroxidase, APX = ascorbate peroxidase, GR = glutathione reductase, ASC = ascorbate, MDHAR = monodehydroascorbate reductase, MDHA = monodehydroascorbate, AH_2_ = oxidizable substrate, DHA = dehydroascorbate, DHAR = dehydroascorbate reductase, POX = non-specific peroxidase, GSH = reduced glutathione, GSSG = oxidized glutathione. Adapted from [54].

The enzymatic antioxidant system is made up of interrelated antioxidant enzymes including superoxide dismutase (SOD), ascorbate peroxidase (APX), catalase (CAT), glutathione peroxidase (GPX), peroxidase (POX) and glutathione reduction (GR) [47,48]. On the other hand, the non-enzymatic antioxidant machinery is made-up of low molecular weight compounds such as phenolic compounds, amino acids, carotenoids, glutathione (GSH), ascorbic acid and α-tocopherol, which serve important roles in the detoxification of ROS. Apart from their antioxidant properties, these low molecular weight compounds can also function as osmoprotectants under abiotic stress, particularly under drought stress [49,50,51]. Furthermore, the antioxidant machinery (non-enzymatic and enzymatic) functions synergistically for stress alleviation or to enhance the plant tolerance to abiotic stress conditions (Figure 2) [46,52]. For instance, the ascorbate-glutathione cycle (also known as the Halliwell-Asada pathway) functions alongside enzymes such as CAT, GR and APX in the high capacity redox-homeostatic H_2_O_2_-scavenging pathways [53]. In this cycle, APX catalyzes the reduction of H_2_O_2_ to water, utilizing ascorbate (ASC). This reaction then yields monodehydroascorbate (MDHA), which is fated to dismutate to ASC and dehydroascorbate (DHA) or be reduced to NADP^+^ via monodehydroascorbate reductase (MDHAR). Furthermore, dehydroascorbate reductase (DHAR) reduces DHA to ASC using GSH as the reducing substrate. This reaction yields oxidized glutathione (GSSG), which is then converted back to the reduced state (GSH) by NADPH via the action of GR. ASC and GSH take part in the cyclic transfer of reducing equivalents without being consumed in the reactions, thus allowing the scavenging of H_2_O_2_, with NADPH as the reducing equivalent donor (Figure 2) [54].

Recent studies have begun to reveal molecular intersections and biochemical networks between biotic and abiotic stress responses, as well as overlapping regulatory mechanisms in combined stress responses [20,55,56]. A detailed account of plant responses to combined abiotic and biotic stresses is beyond the scope of this review, but the reader is referred to the literature cited herein for more details. However, this observation (i.e., combination of stresses) points to the reality of plants in their natural environments, where the plants interact with both abiotic and biotic stresses, individually or simultaneously, and in the presence of microbial communities (composed of bacteria, fungi and oomycetes) that inhabit the host plant, soil and surroundings, often in symbiotic relationships [57,58]. Some of these beneficial microorganisms found in the rhizosphere have been shown to induce systemic defense and tolerance mechanisms or precondition the plant protective responses against a range of environmental stresses.

### Priming against Abiotic Stresses

Plant priming is a natural phenomenon, described as potentiating the protective and defensive responsiveness of plants upon the perception of some signals from the environment. Studies have demonstrated that plant priming—the preconditioning of plant defense mechanisms by beneficial microbes or agrochemicals—results in a faster, stronger and effective defense and resistance response to environmental stresses [59,60]. Mechanistically, priming can be described as a systematic multistage process consisting of three main stages, namely: (i) the priming phase, (ii) the post-challenge primed state and (iii) the transgenerational primed state. Some of the essential metabolic events observed during the priming phase include the biosynthesis or increase in the levels of amino acids, hormone conjugates and sugars, and these metabolic changes are collectively referred to as the “priming fingerprint” in the priming phenomenology [4,15]. These molecular and cellular changes are stored in the form of “metabolic memory”, “molecular memory”, “stress memory” or “primed memory”’, which can be described as “sensitization” or “immunization” strategies of plant defense systems. Upon a subsequent (or secondary) challenge—the post-challenge primed state—the “primed” plant effectively launches rapid and stronger defense responses [61,62]. The metabolic reprogramming observed during this phase mainly involves changes in secondary metabolite levels—i.e., defense-related compounds such as phenylpropanoids, terpenoids, volatiles, glucosinolates and tryptophan-related metabolites [4,63].

Emerging studies have demonstrated that various priming agents/stimuli can be applied to sensitize the plant defense system against abiotic stresses. For instance, parental drought priming has been shown to enhance drought tolerance in wheat offspring via increased accumulation of proline and glycine betaine [64]. Relatedly, silicon (Si)-based biostimulants have been shown to prime wheat seedlings against salt stress by reducing the accumulation of sodium ions, thus improving salt stress tolerance [65]. In another study by Zhang [66], drought priming was also shown to potentiate the defense response against heat stress in tall fescue (*Festuca arundinacea*) via lipidome reconfiguration. Furthermore, Shehu et al. [67] reviewed the priming effects of β-aminobutyric acid (BABA) against abiotic stresses such as salt stress, drought, nutrient stress and heavy metal stress; some of the general physiological, biochemical and molecular alterations potentiated by BABA included improved stomatal regulation and photosynthesis, water use efficiency, cell membrane remodeling, enhanced ROS detoxification and stress-related gene expression. The molecular and cellular events underlying the priming phenomenon may differ and/or overlap depending on the priming stimuli and the secondary stress [68]. Despite the exponentially increasing efforts to elucidate the metabolic changes that define the priming events in time and space, there is still much to uncover in order to understand this potentiation of plant protective defenses. The comprehensive molecular and biochemical networks involved in the establishment of priming stages are still far from being fully characterized. Regardless of the shortcomings and limitations as stated above, defense priming is undoubtedly one of the key adaptive strategies employed by nature. Thus, this preconditioning of plant defense systems is considered a prospective and complementary alternative means that offers new avenues for plant resistance against environmental stresses.

## 3. Biostimulants as Agronomic Tools to Promote Plant Growth and Counteract Abiotic Stresses

In the last decade, there has been exponentially-growing attention to plant biostimulants as a potential solution to mitigate the negative impacts of the changing climate on agriculture, and becoming one of the pillars of a new agricultural revolution for sustainable food production. Even though the concept of biostimulants surfaced in 1933, only in more recent years has attention and studies from different fields emerged to define, describe and understand plant biostimulants, as well as their modes of action [10,69]. Historically, the definition of “biostimulants” has evolved and been reformulated over the years, although it has often been poorly described due to a lack of a scientifically-based theoretical foundation for the conceptualization and characterization of these materials that show potential in improving plant health and development. The recent review by Yakhin et al. [10] provides a detailed account and a chronological evolution of the concept of the term biostimulant. Currently, a biostimulant is conceptually defined as any substance or microorganism that is not a nutrient, pesticide or any of the soil improvers, but has the ability to promote the health and growth of a plant through the induction of natural biological processes [10,70,71].

### 3.1. The (Lack of) Science of Biostimulants

Most plant biostimulant products currently on the market are based on claims and descriptions of the observed effects. However, such descriptions or characterization do not demonstrate if a product is indeed a bona fide biostimulant, guaranteeing a specific level of efficacy and effectiveness under all conditions [10,71]. Hence, there is a growing need for a sound scientific foundation to pave a theoretical framework that would contribute to the formulation of biostimulant products, with scientifically-based descriptions and credibility. Although there is currently no worldwide regulatory system and harmonized legislative framework for the biostimulant industry, there is a momentum in fundamental scientific research, beyond agronomic traits assessment, to informatively generate knowledge and understanding of plant biostimulants, their biological and chemical characteristics and decoding the complex plant-biostimulant interactions. Such insights provide a relatively standardized foundation for guidelines for biostimulant products and have been influential in the development of a European Union regulation and legislation system for biostimulants [10,70]. 

Furthermore, recent scientific meetings in the field of biostimulants have greatly contributed to the conceptual and methodological development, as well as promotion of scientific principles in biostimulant theory and applications. The recent review paper by Ricci et al. [71] highlights the ongoing efforts to advance the scientifically-based biostimulant industry and describes the type of informational data that are prerequisite to support a biostimulant claim. Credible published literature that is of great quality, detailing the mechanisms of action employed by a biostimulant and the use of experimental data obtained from either a greenhouse/controlled environment or field trials can be used to support a claim. However, there are strict guidelines governing the nature of the experimental data that can be admissible as evidence. For instance, for a field trial, the following details must be provided: (i) the specific objectives of the trial, the expected effects of the biostimulant, as well as the application rate and method; (ii) site specifications (i.e., location address and climatic conditions) and type of crop and cultivar; (iii) statistical analysis and trial design—i.e., adequate amount of control groups is required to accurately attribute all the observed distinct features to the treatment applied; and (iv) the trial conditions (i.e., row spacing, type of soil, etc.). The Natural Sciences Information Centre’s Biostimulants online (https://biostimulants.online/) is the largest database of published experimental data on plant biostimulants, which relies on accurate, detailed information from the authors. Additionally, for more details on the global perspective on the biostimulant field, the reader is encouraged to refer to the recent review paper by Yakhin et al. [10].

Plant biostimulants are particularly useful in organic farming, where these substances can aid in overcoming nutrient limitations by improving nutrient availability, uptake and assimilation [72]. However, the best results can be expected when biostimulants are added to well-managed soils in addition to chemical fertilizers. While a vast number of commercial plant biostimulants are available on the market, there is still an element of unpredictability with regard to the effect it might have on a crop, as this varies based on the soil, environmental factors, application rate and time, species and even cultivar [73]. This points to the knowledge gaps in understanding the mechanisms of plant biostimulants at cellular and molecular levels. Some results of plant trials using commercial plant biostimulants are summarized in review papers [73,74]. However, it suffices here to highlight some of the key mechanisms employed by different classes of biostimulants in plant-biostimulant interactions.

### 3.2. The (Bio)Chemical Mechanisms Mediated by Different Classes of Biostimulants

Plant biostimulants can be classified into two main groups: (i) microbial biostimulants such as plant growth-promoting rhizobacteria (PGPR) and fungi; (ii) non-microbial biostimulants such as seaweed extracts, humic substances, protein hydrolysates (PH), chitosan and other biopolymers [70,75]. Observed and elucidated effects of biostimulants include increasing the rate at which the plant absorbs and assimilates the nutrients and improving the quality traits of crop plants [70,74]. Furthermore, studies have shown that the application of biostimulants can enhance the plant immune system, leading to resistance against pathogenic diseases and tolerance against abiotic stresses [75,76,77]. 

Microorganisms have a role to play in stress tolerance. An important consideration in the development of microbial inoculants is the commercial formulation—i.e., the microorganisms should be able to survive in the formulation [78]. Bacteria, belonging to several genera (*Rhizobium*, *Bradyrhizobium*, *Azotobacter*, *Azospirillum*, *Pseudomonas* and *Bacillus*) and with the potential to act as biostimulants have been isolated from saline, alkaline, acidic and arid soils. Members of these genera have developed strategies to adapt under adverse conditions which include alterations to the composition of the cell wall (exopolysaccharide production which forms a protective biofilm on the root surface) and the ability to accumulate high concentrations of solutes, thus allowing for enhanced water retention and increased tolerance to ionic and osmotic stress. PGPR-inoculated soils can ameliorate plant abiotic stress responses [75]. Growth enhancement is usually associated with high levels of IAA, commonly associated with salt stress alleviation [79], as well as exopolysaccharide production that may help in maintaining a film of hydration around the roots. The protective effects of biostimulants on plants that are exposed to environmental stresses such as water deficit, soil salinization and sub-optimal growth temperatures are of vital importance for the survival of crops. This tolerance can also be brought about by microbes within and around the plants that facilitate adaptation to abiotic stress.

A substantial amount of seaweed is also used as a nutrient supplement and plant biostimulant; by 2006 it was estimated at 15 million ton per annum according to a report by FAO (2006). These are applied as soil conditioners or as plant stimulators as a foliar spray to enhance plant growth as well as freezing, drought and salt tolerance, photosynthetic activity and resistance to fungi, bacteria and virus, thus improving the productivity of many crops [80]. The biostimulant effects have been attributed to the presence of plant growth hormones (e.g., cytokinins) and low molecular weight compounds present in seaweed extracts [81]. Furthermore, the study by Bradáčová et al. [82] revealed that the application of seaweed extract-based biostimulants containing zinc and manganese to maize crops can improve the cold resistance/tolerance via enhanced ROS scavenging systems. In another study by Desoky et al. [83], biostimulants derived from ASC and *Moringa oleifera* leaf extract (MLE) were shown to improve salt stress amelioration in pea plants by increasing antioxidant enzymes (CAT, POX and SOD), sugar and proline.

Humic substances were traditionally considered to be polymers, but that has recently been challenged. The alternative is that humic substances are supramolecular associations of heterogeneous, relatively small molecules [84]. Humic substances positively affect plant growth by forming soluble complexes with micronutrients which reduces leaching thereof, thus making them more available to the plant [85]. It has furthermore been reported that intact micronutrient cation-fulvic acid complexes were taken up by the plant due to the low molecular size of the latter [86]. With regard to abiotic stress, humic acid and phosphorus applied to bell peppers resulted in plants with reduced Na content and elevated N, P, K, Ca, Fe, Mg, S, Mn and Cu ions in roots and shoots [87]. Humic acid extracted from vermicompost also exhibited benefits to rice in activating anti-oxidative enzymatic function and increased ROS scavenging enzymes [88]. These enzymes are required to inactivate toxic free oxygen radicals produced in plants under drought and saline stress. It should be noted that the application rate is of vital importance; in a hydroponic study inhibition of shoot growth of maize was observed [89]. 

PH impact plant nutrition by forming complexes and chelates between soil micronutrients such as Zn, Fe, Cu and Mn, thereby aiding in nutrient availability and uptake in the roots [70,90]. Hydrolysates show antioxidant and free radical scavenging properties, in addition to their chelating abilities [91]. PH work by improving microbial biomass and activity as well as soil respiration, since microorganisms can utilize amino acids and peptides as sources of C and N [92]. Maize plants under salt stress treated with hydrolysate-based biostimulants containing triacontanol and indole-3-acetic acid had higher flavonoid, proline and K content over untreated controls [93]. Similarly, with lettuce under salt stress, plant-derived protein hydrolysates improved the fresh yield, dry biomass and root dry weight as well as increased concentrations of osmolytes, glucosinolates and the composition of sterols and terpenes [94]. Glycine betaine is known to accumulate in plants in response to abiotic stresses [95]. Glycine betaine and proline have been shown to increase tolerance for environmental stresses such as freezing, salinity, drought and oxidative stress [70].

Silicon similarly acts by minimizing metal and metalloid toxicities through the complexation or co-precipitation of toxic metals and metalloids in soil and plant tissue, as well as stimulation of antioxidant systems in plants [96]. These authors also highlighted the important function of silicate in enhancing rigidity, strengthening and elasticity of cell wall. In a study where a commercial biostimulant comprising amino acids, proteins, vitamins and betaines was applied to tomato plants under drought stress, drought responsive genes were enhanced. Furthermore, plants had a higher fresh weight and relative water content [97]. Chitosan possesses antioxidant properties by means of the hydroxylated amino groups on the oligomers, making these compounds effective scavengers of hydroxyl radicals and anion superoxide [98]. Chitinases are also considered important in plant resistance to several abiotic stresses. For example, chitosan treatments had a significant effect on the yield of drought-stressed plants compared to a control. This effect was greatest when chitosan was applied before the onset of stressful conditions [99]. This observation was corroborated by researchers investigating the improved osmotic potential tolerance in safflower plants [100]. It was furthermore found that this only holds true at low concentrations, since it is hypothesized that high concentrations cause an absorptive obstruction due to the stickiness of chitosan. The cationic properties of chitosan make it suitable as a carrier for other essential elements (Cu, Fe, Zn, etc.). Certain horticultural and agricultural crops have been genetically engineered to express foreign chitinases to provide resistance to pathogens and abiotic stress [101]. More examples of the functional and mechanistic roles of biostimulants are provided in Table 1.

These previous studies have demonstrated that the application of biostimulants promotes plant growth and development and can alleviate abiotic stresses, enhancing plant responses via different biological and physiological processes that include ROS scavenging mechanisms, membrane stability, osmoprotection, stomatal regulation and xylem hydraulic conductance, root zone water and nutrient availability, metal chelation and changes in hormonal levels [75]. However, fundamental research that provides an integrated view of cellular and molecular mechanisms underlying biostimulant–plant interactions is still enigmatic. Further research on the components and effects of biostimulants on the plant genome and cellular metabolism is required to elucidate the mechanisms of action involved in growth responses and stress mitigation. Decoding the mechanisms of action of biostimulants at cellular and molecular levels is a prerequisite for the development of a scientifically-based biostimulant industry, thereby leading to an effective exploration and application of biostimulants in agriculture for improved and sustainable food security.

## 4. Omics Sciences to Study Plant Biology in Abiotic Stress Conditions 

**Figure 3:**
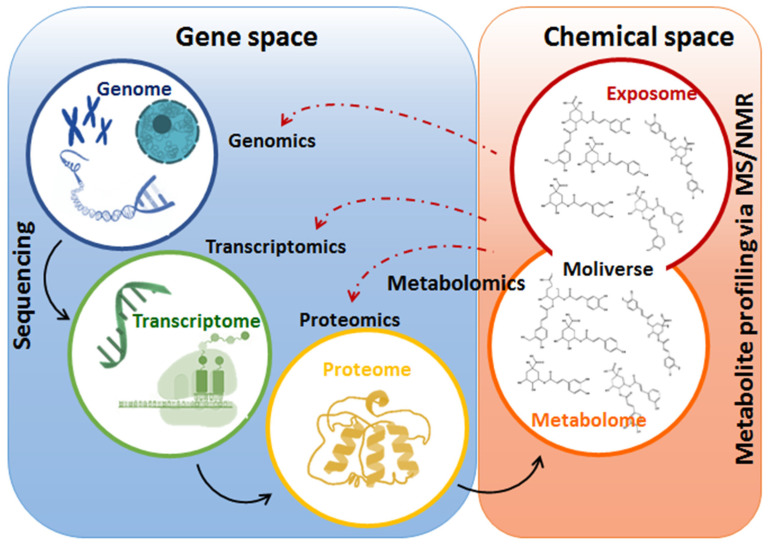
**Summary of the omics disciplines comprising systems biology**. The figure illustrates the flow of biological information from gene to metabolome, highlighting the interconnection of gene and chemical spaces of a biological system. In a stressful event, gene expression is altered, and chemical spaces are reprogrammed to maintain cellular and molecular life-defining equilibria. At the metabolome level, changes in the levels of primary and secondary metabolites play an important role in the readjustment of cellular metabolism towards adaptation to various environmental conditions.

The application of omics approaches such as genomics, transcriptomics, proteomics and metabolomics (Figure 3) have proven to be instrumental in the investigation and identification of the key multilayered biochemical events and mechanisms underpinning the effects of biostimulant formulations on plants’ physiology. For instance, a previous transcriptomics study reported that the use of a novel technology, next generation sequencing (NGS), allowed the monitoring of the impact of biostimulants on the transcriptome of plants, thus revealing the molecular mechanisms of action by which the biostimulant enhanced growth promotion in corn and soybean. The mechanisms of action elucidated in corn included the upregulation of nitrogen and phosphate assimilation and metabolism, maltose biosynthesis, sugar transport and phloem loading and hormone (cytokinin) metabolism, whereas in soybean, growth enhancing metabolic processes that were upregulated included nitrogen metabolism, sulfate reduction, metal and ion transport and amino acid biosynthesis [111]. 

As exemplified above, transcriptomics studies can contribute to the ongoing scientific efforts to provide fundamental research insights that describe the mechanisms and modes of action of biostimulants. However, these studies present challenges such as the fact that an increase in the levels of mRNA does not always correlate with protein levels and not all the translated proteins are enzymatically active. Furthermore, the outcome of the transcriptome and proteome profiling can be limited by the identification of mRNA and proteins which depends on organism-specific genome information. Thus, due to these limitations, changes in the transcriptome or proteome level do not necessarily correspond to the alteration in biochemical phenotypes and may not accurately reflect the biochemical status of the plant in response to abiotic stresses and biostimulants [112]. Thus, an integration of upper omics levels in the systems biology, with metabolomics studies (Figure 3), can provide a holistic and comprehensive understanding of the molecular mechanisms of actions mediated by biostimulants to mitigate abiotic stresses.

### 4.1. Metabolomics in Plant Biostimulant Studies

Metabolomics, a holistic qualitative and quantitative study of the entire complement of metabolites within a biological system, has disruptively positioned itself as one of the central pillars in systems biology and increased in popularity and applicability across a vast array of fundamental as well as translational research domains [13,113]. This distinct position of metabolomics among the modern omics disciplines in the systems biology approach arises from the fact that the metabolome is the cornerstone of life, composed of metabolites which are the final recipients in the flow of biological processes. Furthermore, the analysis of the metabolome can reflect on both transcriptional and post-transcriptional regulation of gene expression, thus being the closest omics approach to the phenotype characterization [8,14]. 

#### 4.1.1. An Overview of the Metabolomics Workflow

As a fast-growing fundamental omics discipline with many advantages, researchers have also identified various shortcomings that limit the extraction of the essential biological information from metabolomics studies. Thus, numerous efforts aimed at maximizing metabolomics outputs are being made, from the very initial step, “study design” to the last step “biological interpretation”. Prior to the beginning of any metabolomics study, it is imperative to formulate a clear, concise and specific biological question/hypothesis that will guide the design of an experimental procedure, which is equipped to adequately yield maximum useful information. Failure to formulate a clear biological question/hypothesis can lead to misinterpretation or multiple possible interpretation of the metabolomics results, thus failing to provide insights or accurately answer any question at all in regard to the biological system under consideration [114]. An ideal experimental design should consist of the smallest number of experiments that can yield the maximum amount of data and achieve accuracy and precision, whilst addressing sample size effect (treatment groups and control groups), as well as taking into account the confounding variables and factors [115]. Recent reviews by Ivanisevic and Want [116] and Rodrigues et al. [115] provide some useful guidelines that should be considered at the experimental drawing board; thus, readers are referred to these citations for detailed descriptions.

**Figure 4:**
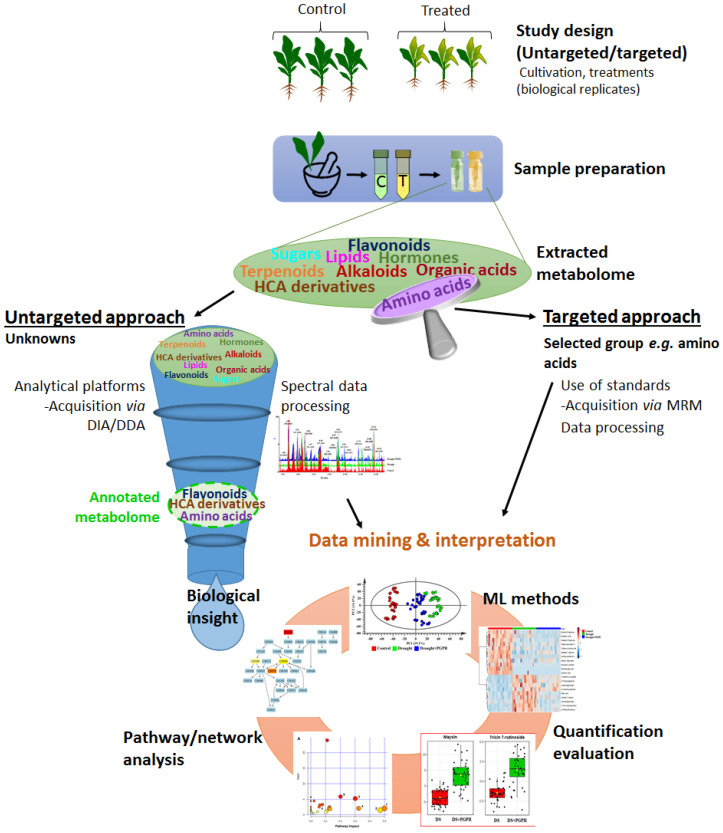
**A schematic representation of a metabolomics workflow.** The infographics depicts key components in the multistep mass spectrometry-based (plant) metabolomics workflow pipeline, which include study design (untargeted/targeted), sample preparation, data acquisition, mining and interpreting metabolomics data. Abbreviations: DIA = data independent acquisition, DDA = data dependent acquisition, ML = machine learning.

Moreover, one is required to carefully choose a metabolomics approach suitable to address the biological question at hand. Metabolomics approaches can be untargeted and targeted (Figure 4). Untargeted metabolomics aims to unbiasedly measure all detectable metabolites, thus providing a comprehensive global analysis of the metabolome [116,117]. The targeted approach allows the quantification of a pre-defined small specific group of metabolites. Moreover, untargeted approaches are applied in hypothesis-generating studies, while targeted approaches are usually applied to further confirm, elaborate and interpret the novel findings of hypothesis-generating studies [118,119]. Arguably, metabolomics is the most challenging and demanding of the omics fields due to the ontological complexity of the metabolome—the “moliverse” of cellular worlds (Figure 3 and Figure 4). This observation points to the challenges found at different levels of the workflow pipeline, limiting the biological insights generated from a metabolomics study (Figure 4). These bottlenecks include metabolome coverage (affected by both the metabolite extraction method and analytical tools), extraction of useful information from raw data and data interpretation and integration of orthogonal biological information from other omics fields.

Several reviews have presented and discussed the metabolomics workflow in general, and/or aspects of metabolomics studies [13,120]. It suffices here to mention that the general plant metabolomics workflow comprises multiple steps, of which the main stages include (i) study design, (ii) sample preparation, (iii) data acquisition, (iv) data processing and (v) statistical analysis and interpretation (Figure 4). As a multidisciplinary omics science, a metabolomics study is a team effort, implying a spectrum of expertise including analytical chemistry, chemometrics, data science and field-specific biology [16,121]. An appropriate study design, incorporating feedback from the entire team, is essential in obtaining high quality data. Addressing a specific biological question (regardless of whether the study is an inductive or deductive one) typically impacts sample preparation, analytical methods and statistical analysis along with a biological interpretation of the “finished product” [120]. Pre-analytical steps in a metabolomics workflow include generation of the samples (Figure 4), and their preparation for analysis. The latter includes plant material harvesting/collection, metabolite extraction and any other processing for analysis. Considering the sensitivity of the metabolome, uniform and consistent sample handling is essential to minimize experimental error. Additionally, to maintain the stability of metabolites and to avoid degradation (or any unwanted enzymatic reactions), quenching metabolism via the use of liquid nitrogen is recommended to arrest the metabolism (during harvesting and metabolite extraction) [115]. Any processing of biological samples provides a window for the introduction of biologically irrelevant variations, thus considered one of the primary sources of bias in the metabolomics data. Randomization during sample preparation and the analytical process is also important to minimize systematic bias [115,120] and effective quality control and quality assurance measures should be put in place to provide evidence of experimental rigor [122,123]. For validation and credibility of plant metabolomics studies, strict measures such as following the Metabolomics Standard Initiative (MSI) has been set in place, proposing the minimum requirements at different aspects of metabolomics workflow and domains [124].

Moreover, the plant metabolome is very complex and diverse, consisting of metabolites of heterogeneous nature with respect to varying physical and chemical properties such as size, polarity, chemical stability, volatility, solubility and quantity, thus rendering the analysis of the entire metabolome close to impossible [13,125]. Therefore, for the untargeted metabolomics approach, the choice of the metabolite extraction method is crucial to obtain maximum metabolome coverage [115,126,127,128]. Over the years, the thumb rule, “like dissolve like” extraction techniques have been among the general methods employed in metabolomics investigations. These techniques involve the use of various organic solvents, whereby polar solvents are used to extract polar metabolites, and nonpolar solvents are used to target the nonpolar constituents from the plant matrices [129,130]. Generally, most researchers prefer to use a medium where solvents such as 80% methanol are used to target a wider range, mid-polar to non-polar metabolites. However, this kind of approach still does not reflect a true representation of the entire plant metabolome. Thus, methods such as parallel/direct and sequential extraction approaches have been explored to enhance the metabolome coverage. Parallel extraction techniques involve the extraction of metabolites using various extraction methods such as solid phase extractions, solvent precipitation and liquid-liquid extraction, of which the extracts from each method are analyzed (e.g., on LC-MS) separately. The combination of these extraction techniques has been shown to increase the metabolome coverage and has been selected to be orthogonal; however, it is not a practical solution since it is laborious and increases the analytical time. Sequential extraction, on the other hand, has been reported to enhance the metabolome coverage at a minimal expense [131]. In this approach, extracts from each extraction method (done in a serial manner) are pooled together before the analysis, thus minimizing the data acquisition time [132].

The analytical step in the metabolomics workflow is the acquisition of data using analytical techniques such as chromatography-mass spectrometry (GC-MS, LC-MS), nuclear magnetic resonance (NMR) spectroscopy, capillary electrophoresis (CE), direct infusion-MS (DIMS), Fourier-transform infrared (FT-IR) and Raman spectroscopy, with each offering differential levels of selectivity, sensitivity, speed, accuracy and precision, respectively [118,128]. The choice of the analytical platform may depend on availability of the analytical systems, the biological question to be investigated as well as the nature of the extracted metabolites. To date, there is still no single available analytical technique that is capable of analyzing the whole metabolome [119,133]. A review of orthogonal MS methods for expanding coverage of the metabolome can be found in [134]. Continuous technological advancements in analytical instrumentations are worth appreciating, offering opportunities in detecting more metabolites. MS and NMR are the main analytical platforms in metabolomics. Of recent, MS has been the most preferred data acquisition tool, and approximately 70% of metabolomics papers published in the 2018, reported data acquired through MS-based technologies. MS-based metabolomics has gained such momentum due to several advantages of MS technologies, such as excellent sensitivity, extensive metabolome coverage, numerous software resources availability, small instrument footprint, relatively inexpensive instrumentation and maintenance [135]. 

Although the popularity of MS-based metabolomics seems to overshadow the application of NMR techniques in the metabolomics field, NMR still holds a tremendous promise, as it offers some unique advantages. These include robust instrumentation, precise structural elucidation, easily automated workflow, excellent reproducibility and non-destructive sample preparation. The revolutionary advancements (including the software and hardware) that can be/has been implemented to increase the application and relevance of NMR-based metabolomics are explicitly discussed in the short review by Wishart [135]. The post-analytical step in a metabolomics workflow comprises data mining and interpretation. This multistage step entails data processing, ML and statistical modeling, metabolite annotation and identification, and biological interpretation [13,121]. 

#### 4.1.2. Application of Metabolomics in the Plant-Biostimulant-Abiotic Stress Interaction Studies

The application of metabolomics in plant sciences spans a wide range of domains, including elucidation of metabolic pathways, plant breeding, silent phenotypic mutations, linking genotypes to phenotypes, biotic and abiotic stresses and defense priming [68,125,136]. However, metabolomics applications in the field of plant biostimulants are still very limited. In the last section of this review, we highlight some reports on metabolomics applied in plant biostimulant studies, summarized in Table 2. Currently, there are very few emerging metabolomics studies on the bipartite interaction (biostimulant-plant) and even fewer on the tripartite interaction (biostimulant-plant-abiotic stress). For example, in the metabolomic study by Lucini et al. [94], a protein hydrolysate-based biostimulant alleviated saline stress by increasing the levels of defense-related metabolites (terpenes, amino acids, carbohydrates and sterols) in lettuce. In another study by Arroussi et al. [137], a seaweed extract-based biostimulant (*Dunaliella salina* exopolysaccharides) was applied to tomato plants to investigate its role in the amelioration of saline stress. Using metabolomics, it was confirmed that *D. salina* contains bioactive compounds known as sulfated exopolysaccharides which upregulated the levels of proline, phenolics, osmoprotectants compounds and the activity of antioxidant enzymes, thus leading to salt stress alleviation. Furthermore, the untargeted metabolomics analysis revealed that the enhanced drought tolerance mediated by plant-derived PH in tomato is characterized by key metabolic changes in phytohormones (i.e., accumulation of salicylates and decreased content of cytokinins), in hydroxycinnamic acid amides and lipids such as terpenes, membrane lipids and sterols. Moreover, the observed metabolic reprogramming induced by PH-based biostimulants improved stomatal conductance and redox balancing machinery [11].

Recent applications of metabolomics in studying biostimulant-plant interactions (Table 2) demonstrate the potential of metabolomics as an indispensable (multidisciplinary) tool to investigate the effects of biostimulants on the regulation of plant metabolic networks under abiotic stresses and biostimulant-induced remodeling of complex genetic plants’ traits, providing useful insights for plant breeding and novel agricultural strategies, for sustainable crop production. As mentioned in Section 3, most biostimulant products currently on the market are based on claims derived from phenotypic evaluations. Such descriptions do not demonstrate if a formulation is indeed a bona fide biostimulant, guaranteeing a specific level of efficacy and effectiveness under all conditions. Hence, fundamental insights and an understanding at both cellular and molecular levels of modes of action of a biostimulant product is a sine qua non foundation to pave a theoretical framework for the sustainable formulation of biostimulant products. 

Thus, as reviewed herein, the current knowledge gaps and major bottlenecks in the biostimulant industry include (i) the lack of a universal definition that conceptually captures all essential aspects of biostimulants, (ii) a poor characterization of active components and synergies in the biostimulant composition and (iii) undefined mechanisms and modes of action of biostimulant products, at cellular and molecular levels. Such limitations hinder not only the designing of novel formulations and biostimulant-based agricultural strategies but also the establishment of a standardized legislative framework and regulatory system for the biostimulant industry. Metabolomics, a multidisciplinary omics science, offers unique opportunities to predictively decode the mechanisms and modes of action of biostimulants on crop plants, and elucidating signatory markers and metabolic profiles that define the biostimulant action (Table 2). Application of metabolomics in the biostimulant research can provide a molecular deconvolution of biostimulant-induced remodeling of the plant metabolism towards improved plant health, growth and increased yield. Such fundamental and indispensable knowledgebase would improve our understanding of biostimulants, also providing a roadmap for translational applications—designing of novel formulations and devising biostimulant-based precision agricultural programs and modules—for sustainable food security.

## 5. Concluding Remarks

This review provides a general overview of the current knowledge regarding plant defense mechanisms, the definition and the application of biostimulants in agriculture, and lastly, gives a review of the application of metabolomics approaches used to study the interactions between plants, biostimulants and abiotic stresses. Plant growth and resistance against abiotic stresses have long been the quest of crop breeders; thus, in recent years, various methods such as genetic engineering, traditional and modern breeding techniques have been developed and applied to support this endeavor. Furthermore, agronomic management strategies, such as frequent irrigation and grafting, have also been devised and implemented to assist plants in coping with adverse environmental constraints. However, these approaches and agricultural programs have limitations, and some of the agricultural practices carry high environmental costs. Thus, a new revolution such as the use of biostimulants is inevitably needed; the global food and nutrition security need imperatively more and new science to sustain a growing global population and a changing climate. However, there are still many grey areas that need to be defined to holistically understand the complexity of the plant–biostimulant–abiotic stress relationship. As such, metabolomics studies can contribute towards the continuing efforts in identifying and defining the multifaceted biochemical and molecular processes induced by biostimulants in crop plants, improving growth and enhancing plant immunity. 

## Figures and Tables

**Table 1:** **The effects of biostimulants on crop productions**. The table provides some examples whereby cellular and physiological mechanisms underlying the effects of the selected biostimulants under various stresses were demonstrated as well as their agricultural/horticultural functions and the expected economic and environmental benefits (adapted from du Jardin [70]).

Main Biostimulant Classes	Cellular Mechanism (i.e., Interaction with Cellular Components and Processes)	Physiological Function (i.e., Action on Whole-Plant Processes)	Agricultural/Horticultural Function (i.e., Output Traits Relevant for Crop Performance)
**Humic substances**	Activate plasma membrane proton-pumping ATPases, promote cell wall loosening and cell elongation in maize roots [102]. Increases antioxidation capacity under several abiotic stresses, upregulates the biosynthesis of defense-related secondary metabolites [103].	Increased linear growth of roots, root biomass [103].	Increased root foraging capacity, enhanced nutrient use efficiency [103,104].
**Seaweed extracts**	Stimulates expression of genes encoding transporters of micronutrients (e.g., Cu, Fe, Zn) in *Brassica napus* [105].	Increased tissue concentrations and root to shoot transport of micronutrients	Improved mineral composition of plant tissues
**Protein hydrolysates**	Stimulation of phenylalanine ammonia-lyase (PAL) enzyme and gene expression, and production of flavonoids under salt stress [93].	Protection by flavonoids against UV and oxidative damage [106].	Increased crop tolerance to abiotic (e.g., salt) stress
**Chitosan**	Induce the accumulation of reactive oxygen species (ROS) and phenolic compounds. enhances the ROS scavenging system, regulates the stomatal conductance [107].	Increased all vegetative growth and yield [108].	Protection against brown rot, delayed fruit softening and senescence [109].
**Microorganisms (e.g., PGPR)**	*Azospirillum brasilense* releases auxins and activates auxin signaling pathways involved in root morphogenesis wheat [110].	Increased lateral root density and surface of root hairs	Increased root foraging capacity, enhanced nutrient use efficiency
**Fungi**	Enhance phosphate (P) availability under nutrient deficiency via excretion of P-solubilizing substances [72]	Increased root growth and activity	Increased nutrient availability in the soil

**Table 2:** Some applications of metabolomics in elucidating mechanisms and modes of action of plant biostimulants.

Study	Biostimulant	Effects	Plant	Mechanism of Action—Metabolic Changes	References
*Capsicum chinensis* L. growth and nutraceutical properties are enhanced by biostimulants in a long-term period: Chemical and metabolomic approaches	Plant-based biostimulants: one derived from AH and RG	Growth promotion	Pepper	− Production of secondary metabolites, such as phenols.	[138]
The effect of a plant-derived biostimulant on metabolic profiling and crop performance of lettuce grown under saline conditions	Plant-derived protein hydrolysates	Growth promotion and resistance to salt stress	Lettuce	− Improved plant nitrogen metabolism − Oxidative stress mitigation via the increase in osmolytes, changes in sterols, glucosinolates and terpenes composition	[94]
*Dunaliella salina* exopolysaccharides: a promising biostimulant for salt stress tolerance in tomato (*Solanum lycopersicum*)	Microalgal exopolysaccharides	Resistance to salt stress	Tomato	− Activation of jasmonic pathway − Accumulation of VLCFAs to strengthens the plant’s cuticles − Inactivation of SA pathway − Increase in phytosterols to regulate membrane fluidity − Induction of ROS scavenging enzymes and other antioxidant molecules	[137]
Effects of humic substances and indole-3-acetic acidon Arabidopsis sugar and amino acid metabolic profile	Humic substances	Growth promotion	Arabidopsis	− Possible increased activity of glycolysis − Reduced free amino acids, suggesting increased protein and/or secondary metabolites production	[139]
A vegetal biopolymer-basedbiostimulant promoted root growth in melon while triggeringbrassinosteroids and stress-related compounds	Vegetal biopolymer-basedbiostimulant	Growth promotion	Melon	− Changes of brassinosteroid content was linked to increased root development, as well as the modulation of photosynthetic activity − Interference with hormonal crosstalk in leaves	[140]
Understanding the biostimulant action of vegetal-derived protein hydrolysates by high-throughput plant phenotyping and metabolomics: A Case Study on Tomato	Vegetal-derived protein hydrolysates	Growth promotion	Tomato	− Multilayer regulation of ethylene and polyamines metabolism − ROS-mediated signaling pathways	[141]
A combined phenotypic and metabolomic approach for elucidating the biostimulant action of a plant-derived protein hydrolysate on tomato grown under limited water availability	Plant-derived protein hydrolysate	Resistance against drought	Tomato	− Phytohormonal changes (reduced cytokinins and increased salicylates) − Changes in lipids and terpenes − Oxidative stress mitigation via hydroxycinnamic amides, carotenoids, prenyl quinones, and reduced biosynthesis of tetrapyrrole coproporphyrins.	[11]
Metabolomic analysis of the effects of a commercial complex biostimulant on pepper crops	Commercial biostimulant, Actium	Growth promotion	Pepper	− Increased phenylalanine and total monosaccharides, suggesting a further stage in ripening. − Increase in carotenoids concomitant with an increase in some digalactosyl diacylglycerols	[142]
Effect of microalgae polysaccharides on biochemical and metabolomics pathways related to plant defense in *Solanum lycopersicum*	Microalgae polysaccharides	Plant defense	Tomato	− Lipid remodeling − Increased lipids such as VLCFAs associated with thickened cuticular wax	[143]
Biostimulants from food processing by-products: agronomic, quality and metabolic impacts on organic tomato (*Solanum lycopersicum* L.)	Biostimulants from food processing by-products	Growth promotion	Tomato	− Increase in citric acid content and the decrease in *β*-glucose content	[144]
Inoculation of *Rhizoglomus irregulare* or *Trichoderma atroviride* differentially modulates metabolite profiling of wheat root exudates	*T. atroviride* and *R. irregulare*	Growth promotion	Wheat	− Differential changes in lipids, phenolic compounds, terpenoids, siderophores, chelating acids, derivatives of amino acids and phytohormones	[145]
Vegetal-derived biostimulant enhances adventitious rooting in cuttings of basil, tomato, and *Chrysanthemum* via brassinosteroid-mediated processes	Vegetal-derived biostimulant	Growth promotion	Basil, Tomato, and Chrysanthemum	− Biostimulant-derived BRs and auxin were suggested to modulate endogenous BR pool, − Induces morphological and metabolic changes during adventitious rooting of cuttings in plants	[146]
A biostimulant obtained from the seaweed *Ascophyllum nodosum* protects *Arabidopsis thaliana* from severe oxidative stress	Seaweed extracts	Oxidative stress	Arabidopsis	− Accumulation of maltose and fumarate and malate − Reduction in lipids such as TAGs which induces cell death and chloroplast degradation	[147]

Abbreviations: SA = salicylic acid, ROS = reactive oxygen species, VLCFAs = very-long-chain fatty acids, BRs = brassinosteroids, AH = alfalfa plants, RG = red grape, TAGs = triacylglycerols.
